# New optical fibres for high-capacity optical communications

**DOI:** 10.1098/rsta.2014.0441

**Published:** 2016-03-06

**Authors:** D. J. Richardson

**Affiliations:** Optoelectronics Research Centre, University of Southampton, Southampton SO17 1BJ, UK

**Keywords:** optical communications, optical fibres, optical amplifiers

## Abstract

Researchers are within a factor of 2 or so from realizing the maximum practical transmission capacity of conventional single-mode fibre transmission technology. It is therefore timely to consider new technological approaches offering the potential for more cost-effective scaling of network capacity than simply installing more and more conventional single-mode systems in parallel. In this paper, I review physical layer options that can be considered to address this requirement including the potential for reduction in both fibre loss and nonlinearity for single-mode fibres, the development of ultra-broadband fibre amplifiers and finally the use of space division multiplexing.

## 1. Introduction

For the past few decades, communications research has been focused on finding innovative ways of generating, coding, amplifying, (de-)multiplexing and detecting optical signals in order to unlock the enormous potential transmission bandwidth presented by silica-based optical fibres. The identification of silica as the material of choice for optical fibres by Kao and Hockham in the mid-1960s [1] was followed by intensive efforts around the world to realize low-loss guiding structures and following the demonstration of less than 20 dB km^−1^ losses in silica fibres by researchers at Corning laboratories in 1970 [2], there was no looking back. Within less than a decade, fibres with losses of approximately 0.2 dB km^−1^ were demonstrated [3] and the use of single-mode silica fibres to construct high-capacity links for long-haul networks became firmly established.

**Figure 1.**
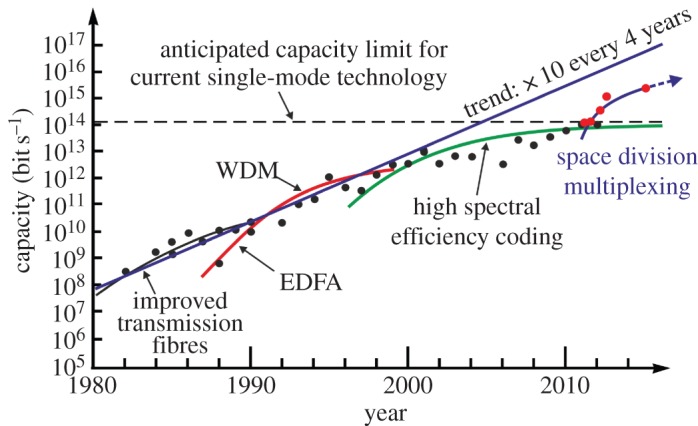
The evolution of transmission capacity in optical fibres as evidenced by state-of-the-art laboratory transmission demonstrations over the years. The data points shown represent the highest capacity transmission numbers (all transmission distances considered) as reported in the Postdeadline Session of the Optical Fibre Communications conference held each year in the USA. The transmission capacity of a single fibre strand is seen to have increased by approximately a factor of 10 every 4 years. Key previous technological breakthroughs include the development of low-loss single-mode fibres, the EDFA, WDM and more recently high-spectral efficiency coding via DSP-enabled coherent transmission (solid red). The data points for space division multiplexing (SDM) also include results from the Postdeadline Session at the annual European Conference on Optical Communications. As can be seen, SDM appears technically capable of providing the next step change in transmission capacity. Adapted from [5], fig. 1.

While significant improvements in standard single-mode fibre (SSMF) performance have been made over the years, as well as in the ability to reliably manufacture such fibres at low cost and in huge volumes (currently at global rates in excess of 200 million kilometre a year), the basic design of SSMF has not changed substantially for many years, and in reality there remains only very limited scope for further optimization [4]. Until recently, the intrinsic capacity of SSMF has been so far in excess of what has been required to meet traffic demands that there have always been far easier and more cost-effective ways to increase link capacity than trying to develop a fundamentally new transmission fibre platform. In most instances, this has been achieved by upgrading the terminal equipment to better exploit the existing available fibre bandwidth. However, as a result of sustained growth in data traffic over many years (typically approx. 40% year-on-year), some of the busiest installed commercial links are now being equipped to run at around 10 Tbit s^−1^ approximately a tenth of the maximum theoretical data capacity of SSMF imposed by optical fibre nonlinearity. Moreover, as highlighted in figure 1, after decades of outstanding human endeavour, and several key technological breakthroughs, we are now within a factor of 2 or so of this fundamental limit in the laboratory. As a consequence, we will likely soon be entering an era in which it will no longer be possible to achieve substantial cost-per-bit reductions simply by changing terminal equipment. In order to accommodate future data traffic growth, it will become increasingly necessary to install new fibres and to light these as required at an essentially fixed cost per added bit. Given this unfavourable economic situation, the question naturally arises as to whether there is anything that can be done to provide more cost-effective routes to capacity scaling by revisiting the whole issue of the optimum underlying fibre transmission medium.

In this paper, I will first review options for increasing the per-fibre capacity beyond that of conventional SSMF technology based on improving single-mode silica transmission fibres and amplifiers. I will then focus the rest of the paper on what currently appears to be emerging as the most promising future approach—specifically the use of space division multiplexing (SDM).

## 2. Options for increasing the per-fibre capacity

Leaving aside for the present some of the underlying physics and engineering issues, we can write the following simple expression for the total capacity *C* of an optical fibre link:
2.1
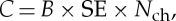
where *B* is the effective transmission bandwidth of the transmission line (accommodating any restrictions due to optical amplifier bandwidth), SE is the spectral efficiency with which we are able to use that available bandwidth and *N*_ch_ is the number of spatial information channels transmitted down the fibre. For existing SSMF technology, *B* is around 10 THz (limited by the bandwidth of the erbium-doped fibre amplifier (EDFA)), SE is the maximum practical spectral efficiency (limited effectively by the optical nonlinearity of the fibre to approx. 5–10 bit s^−1^ Hz^−1^ per polarization) and *N*_ch_=2 (for the two polarization modes associated with a conventional SMF core that are currently used for polarization division multiplexing (PDM)). Using these numbers, we can estimate a maximum total capacity per fibre of between 100 and 200 Tbit s^−1^—in reality, practical/technological/economic constraints are likely to restrict this to around 50 Tbit s^−1^ for commercial systems. In order to increase the per-fibre capacity, we need to be able to increase each of these factors, either individually or preferably in combination.

### (a) Increasing the optical bandwidth

**Figure 2.**
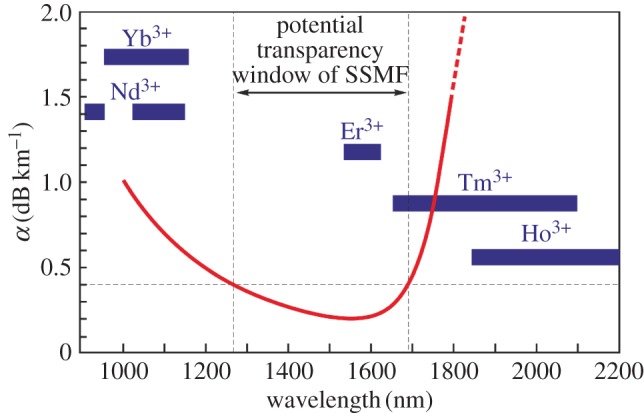
Extending transmission bandwidth. The transmission bandwidth of standard SMF—superposed on which are the amplification bands associated with erbium and other rare earth laser ions incorporated in a silica glass matrix. As can be seen, considerable bandwidth extension would be opened up with the availability of novel amplifiers operating in the range 1300–1530 nm.

In terms of *B*, this essentially means developing combinations of optical amplifier and transmission fibre offering increased net bandwidth. In figure 2, we plot the transmission bandwidth of silica fibre along with an indication of the approximate usable gain bandwidths associated with the various rare earth ions that have been shown to provide good optical amplifier performance, and practical device implementations, when incorporated in a silica-based glass host. Several things can be taken from this figure: firstly, it confirms the fact that it is the bandwidth of the EDFA (approx. 10 THz for the C+L bands) that defines the effective bandwidth constraint for current SSMF technology rather than the intrinsic spectral loss profile of SSMF itself, which can in principle support reasonable transmission (loss less than 0.4 dB km^−1^) from wavelengths just below 1300 nm out to around 1700 nm (corresponding to a potential bandwidth of more than 50 THz). In fact, in practice, current commercial systems only use C-band EDFAs and thus exploit only approximately 5 THz of the available SSMF bandwidth. There currently appears to be little interest among operators to use the L-band, although this may well change as the economics of building networks evolves as this provides a relatively ready route to an additional approximately 3 dB capacity for many existing installed links. Secondly, it can be seen that other than erbium, no other rare earth ion provides good optical amplification within the low-loss transparency window of SSMF when incorporated in a silica glass host. Ytterbium (and also neodymium) provide good gain performance in the range approximately 950–1120 nm; however, SSMF losses here are more than 0.5 dB km^−1^ and are thus too high for long-haul communications applications, and both thulium and holmium operate at much longer wavelengths where SSMF losses rise rapidly to impractically high levels. For completeness, it is to be noted that potentially useable rare earth ion laser transitions do exist in the low-loss SSMF window if other glass hosts are considered, e.g. tellurite and fluoride glass hosts. However, amplifiers based on these glasses suffer from a variety of practical issues and these are unlikely ever to be resolved sufficiently to allow ubiquitous commercial deployment. From a transmission fibre perspective, then there remains a potential wavelength window below the C-band from around 1280 to 1530 nm which might in principle be exploited if suitable optical amplifiers can be developed. Bismuth and chromium ions have been shown to offer very wide luminescence bandwidths when incorporated in silica-based glass hosts and thus fibre amplifiers using these dopants are potential contenders to fill this void [6,7]. Bismuth in particular has provided some intriguing results, and although the detailed gain mechanisms are still not fully understood, encouraging amplifier performance has been demonstrated across much of the wavelength range from 1200 to 1530 nm through appropriate choices of pump wavelength, host glass composition and fibre processing conditions. Repeatability of results currently appears a challenge and whether the technology can truly be mastered and practical bismuth-based amplifiers built is yet to be determined. This remains the subject of ongoing research. Raman amplifiers [8], which exploit the intrinsic nonlinearity of silica glass rather than population inversion of laser ions, are another contender. Such devices are capable of providing high gain and reasonable noise performance over significant bandwidths centred at essentially any desired wavelength providing suitable pump sources can be developed. However, while Raman fibre amplifiers have been around for a long time and are commercially viable, there currently appears to be very little enthusiasm from systems vendors to use them at present due to performance, cost, practicality, laser safety and power concerns.

**Figure 3.**
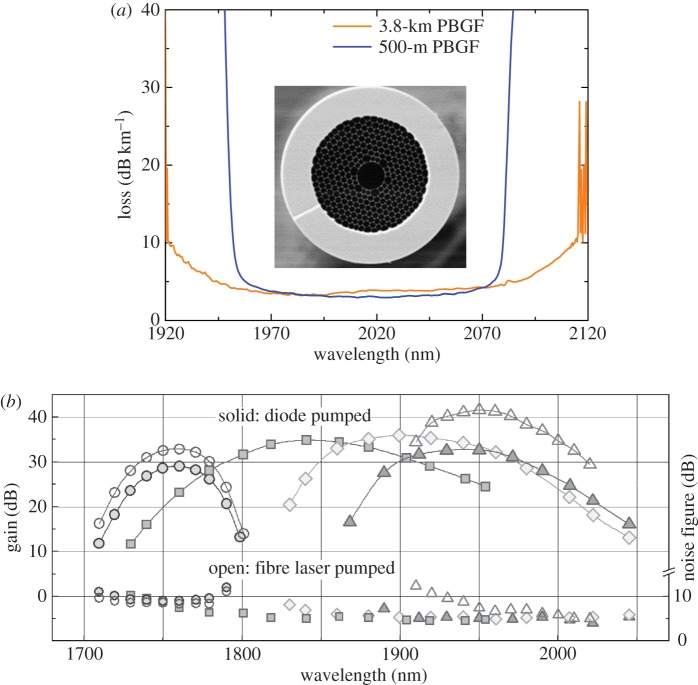
Emerging fibre technology beyond the L-band. (*a*) Spectral transmission profiles for two different HC-PBGF designs of 500 m and 3.8 km lengths, respectively. Inset is a photograph of the cross-section of the 3.8 km HC-PBGF. (*b*) Optical amplifier bandwidth coverage for long wavelength transmission using thulium-doped fibre amplifiers of different designs. Using more advanced designs, bandwidth coverage down to 1650 nm can be achieved. Using holmium-doping wavelength extension out to 2170 nm can be obtained. (Figures (*a*) and (*b*) courtesy of Drs Z. Liu/Y. Chen and Dr Y. Jung, respectively, University of Southampton, UK).

Thirdly, and finally, figure 2 shows that broadband spectral gain coverage above the L-band at wavelengths spanning the range approximately 1700–2050 nm can be realized using the dopant thulium (see figure 3*b* for a plot summarizing recent experimental measurements) [9]. Indeed, through the use of more complex amplifier designs and the additional use of holmium as a dopant, this wavelength coverage can likely be extended from the edge of the EDFA L-band around 1625 nm out to around 2200 nm (with much of this range being accessible with direct diode pumping). In frequency terms, this equates to a bandwidth in excess of 40 THz, three to four times greater than that of the C+L band EDFA. Clearly, this wide amplification bandwidth is incompatible with SSMF due to the strong material absorption of silica glass at these wavelengths. Intriguingly however, there is an emerging class of silica-based fibre, the hollow-core photonic bandgap fibre (HC-PBGF), which guides light predominantly in air rather than glass and for which the minimum loss wavelength is around 2000 nm. The spectral location of the minimum loss window is determined by the combined effects of the scattering of light due to surface roughness at the air : glass interfaces in the vicinity of the core and material absorption due to the 0.1% or so of light propagating in the glass membranes around the core [10]. The predicted minimum loss in these fibres is of the order approximately 0.1–0.2 dB km^−1^, although at present the best measured loss values are still an order of magnitude higher (figure 3*a*). If the ultimate loss/bandwidth potential of HC-PBGF can be realized, then this would open up the potential of exploiting the broad bandwidth of thulium/holmium-doped fibre amplifiers for communications at longer wavelengths around 2000 nm.

Given the above considerations, one might conclude that there is the potential ultimately for a fivefold to 10-fold increase in usable bandwidth relative to the current use of SSMF + EDFAs by developing new amplifiers and/or radically new fibre transmission types—the practical challenges in realizing such an increase though are enormous.

### (b) Increasing the spectral efficiency

**Figure 4.**
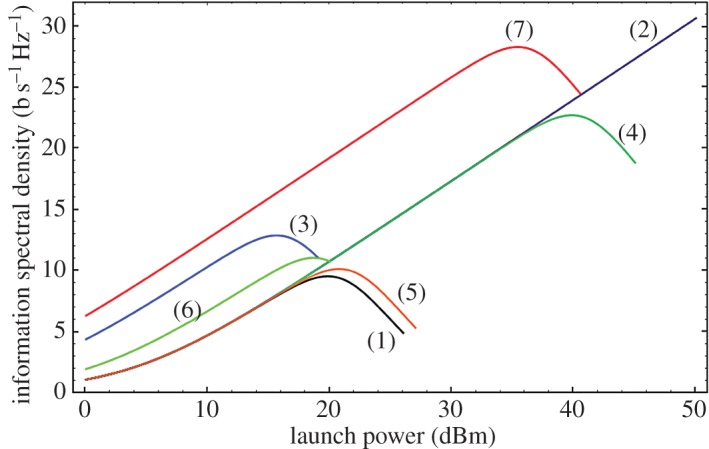
SE benefits of optimizing differenttransmission fibre parameters: theoretical spectral efficiency curves as a function of launched signal power limits for a representative communication system. (1-black line) Baseline parameters of system length = 2000 km, amplifier spacing = 80 km, amplifier noise figure = 4.5 dB, channel spacing = 50 GHz, baud rate = 50 Gbaud, fibre loss = 0.22 dB km^−1^, nonlinearity = 1.4 (Wkm)^−1^, dispersion = 16 ps nm^−1^ km^−1^, wavelength = 1550 nm, bandwidth = 5 THz, number of WDM channels = 101. In the additional plots, all other parameters were kept the same as for the black curve other than (2-indigo line) nonlinearity = 0 (Wkm)^−1^ (conventional Shannon Limit for a SMF system); (3-blue line) loss reduced by a factor of 2–0.11 dB km^−1^; (4-dark green line) nonlinearity reduced by ×1/1000; (5-orange line) dispersion increased by a factor of 2 to *D*=32 ps nm^−1^ km^−1^; (6-light green curve) amplifier noise figure reduced to 1 dB; (7-red line) simultaneous changes (3–6) to the baseline parameters as above. The figure highlights the fact that even by radically improving fibre amplifier properties, then only relatively modest improvements in spectral efficiency can be achieved. It should though be appreciated that the financial benefits of such radical property improvements could be significant. (Figure courtesy of Prof. Andrew Ellis of Aston University, UK).

Substantial work has been undertaken over the past decade looking at optimizing the achievable spectral efficiency in optical communications. This work has focused on exploiting, in combination, the availability of progressively better photonic components (e.g. narrow linewidth lasers, comb sources, modulators, filters, receivers, etc.) and state-of-the-art digital signal processing (DSP) which, by enabling digital coherent transmission, allows full exploitation of the complex optical field for encoding and receiving information. Advanced modulation format signals (QPSK, 16-QAM, 64-QAM, etc.), previously used with such success to achieve high-spectral efficiencies in wireless communications, can now be applied within optical fibre communications. However, it is to be appreciated that there is an important difference between wireless and optical communications imposed by the intrinsic nature of the transmission medium. In wireless communications, the transmission medium has an entirely linear response; however, in optical fibres, the response is intrinsically nonlinear for sufficiently long transmission distances and/or high transmitted power levels and this has an impact on the level of SE that can be achieved in practice. For a linear transmission medium, the maximum achievable spectral efficiency is defined by the well-known Shannon theorem based on fundamental information theory considerations, as given by equation (2.2) and illustrated in figure 4 (line 2—indigo).
2.2
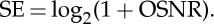
According to Shannon’s theorem, any SE can in theory be achieved simply by increasing the optical signal to noise ratio (i.e. in practice by increasing the signal power sufficiently). However, an optical fibre is inherently a nonlinear medium and the signal power cannot be increased arbitrarily without inducing signal distortions. Any estimate of channel capacity needs to take this into account. In practice, this results in a maximum value of SE that can be achieved for a given system (referred to as the ‘nonlinear Shannon limit’ (figure 4). Various approaches have been proposed in the literature to calculate this limit [11–13]. While there is still debate in the community as to the most appropriate way to do this, we use the analytic approach proposed in [13] to give insight into the sensitivity of the maximum SE to different properties of the transmission fibres and amplifiers used. According to Ellis *et al*. [13]
2.3
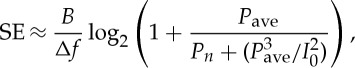
where
2.4
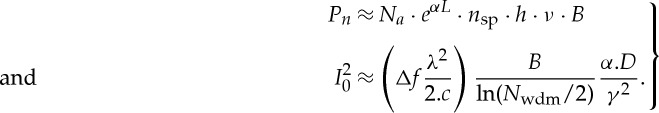
Here, *α*=the fibre loss, *γ*=the fibre nonlinearity parameter, *D*=the fibre dispersion, *c*=the speed of light in vacuum, *λ*=the signal wavelength, *n*_sp_=the spontaneous emission factor (which relates directly to the amplifier noise figure), *B* is the optical bandwidth, Δ*f*=the wavelength division multiplexing (WDM) channel spacing, *hν*=the photon energy, *N*_a_=the number of amplifiers and *N*_wdm_ is the number of WDM channels. It is to be noted that other than optical bandwidth all terms including fibre parameters (i.e. loss, effective area, nonlinearity, dispersion, amplifier noise figure, etc.) lie within the logarithm in equation (2.3)). In figure 4, we plot curves showing how the SE depends on the launched signal power for a representative 2000 km system for different parameter choices (as described in the associated caption). The conclusion to be drawn from figure 4 is that even if we assume fairly gross changes in transmission fibre parameters, then only relatively modest improvements in capacity are possible. For example, even if we move from SSMF to a radically different fibre type such as the HC-PBGF, which offers more than three orders of magnitude reduction in nonlinearity and losses as low as approximately 0.1 dB km^−1^, then this would only provide at most a factor of three to four potential improvement in spectral efficiency. Having said this, it is to be appreciated that a twofold reduction in loss would potentially offer considerable cost-savings in terms of system implementation (e.g. a twofold reduction in the number of amplifiers required) and thus would be of significant commercial interest. From this analysis, then it can be concluded that realistic improvements in single-mode fibres are unlikely to offer significant improvements in spectral efficiency.

### (c) Unleasing the spatial domain

To obtain substantial increases in per-fibre capacity, it is necessary to look at better exploiting the spatial domain since all other physical signalling dimensions (i.e. time, frequency, phase/amplitude and polarization) are already used in current systems and are close to optimally exploited. The spatial dimension is thus the only untapped physical dimension left to turn to [5,14,15]. The idea of increasing fibre capacity by using multiple spatial channels (referred to as SDM) dates back to the earliest days of optical fibre communications with the fabrication of fibres containing multiple cores, a leading contender for SDM, reported as far back as 1979 [16]. The alternative approach of using the individual modes of a multimode fibre (MMF) to define separate spatially distinct channels was itself first proposed in 1982 [17]. The basic premise of SDM is that if we can define *N* distinct spatial channels within a single fibre, then the overall fibre capacity can be increased *N*-fold, or at least close to *N*-fold when any signal degradation mechanisms/mitigation strategies are taken into account.

Interest in SDM has been revitalized due to the rapidly emerging need due to the exponentially increasing traffic demands and concerns of a potential future capacity crunch and has been made technically viable by accumulated advances in fibre research over many years. This includes both the continued refinement of conventional solid fibres (both SMF and MMF variants), as well as the development of the incredibly precise fabrication methods required to produce complex microstructured optical fibres (both solid- and air-containing variants). A further key enabler has been the rapid advances and subsequent commercial adoption of coherent detection and digital compensation which has allowed systems designers to overcome the impact of complex optical impairments in a practical and cost-effective manner. This is crucial since SDM requires the spatial channels to be packed tightly together such that cross-talk between channels becomes a major issue and needs to be reliably addressed. While much can be done optically, e.g. through appropriate fibre design, coherent-detection systems enable the possibility of directly determining cross-talk and of eliminating it electronically at the receiver using DSP.

The promise of SDM is not simply that it will provide the next leap in capacity-per-fibre but rather, and indeed more importantly, that it will facilitate large reductions in cost-per-bit and improved energetic efficiency. The capacity increase benefits of SDM have been quickly and effectively demonstrated in laboratory experiments; however, proving the cost and power consumption benefits represents a far more formidable and ill-defined problem, and much remains to be done on this front. It is to be appreciated that SDM is by its very nature very different from WDM which allows straightforward sharing of key components, e.g. an EDFA and dispersion compensation module can easily be shared by many WDM channels with minimal added complexity, such component sharing is much more difficult to achieve with SDM devices. The commercial benefits of SDM are thus currently more speculative and ultimately assume that many system components can and will be integrated to support cost reduction relative to the use of an array of parallel SMF-based transmission lines of identical net capacity. Moreover, the reliability and performance levels achieved will need to be as least as good as that of existing SMF technology, and the technology will need to provide for a graceful upgrade strategy (i.e. to be capable of operating in a network in conjunction with existing SSMF-based systems).

## 3. Technical approaches to space division multiplexing

**Figure 5.**
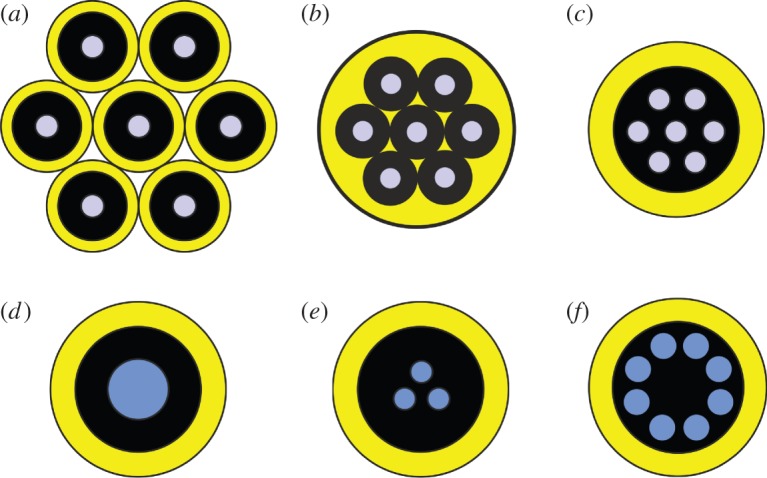
Different approaches to SDM: (*a*) fibre bundle composed of physically independent, single-mode fibres of reduced cladding dimension could provide increased core packing densities relative to current fibre cables; however, ‘in-fibre’ SDM will be needed to achieve the higher core densities and levels of integration ultimately desired. (*b*) MEF comprising an array of independent glass fibres of reduced external diameter coated in a common polymer jacket. (*c*) MCF comprising multiple independent cores sufficiently spaced to limit cross-talk. Fibres with up to 19 cores have so far been demonstrated for long-haul transmission—higher core counts are possible for short-haul applications (e.g. Datacomms) where higher levels of cross-talk per unit length can be tolerated. (*d*) FMF with a core dimension/numerical aperture set to guide a restricted number of modes—so far typically 6–12 distinct modes (including all degeneracies and polarizations). (*e*) Coupled-core fibres support supermodes that allow for higher spatial mode densities than isolated-core fibres. MIMO processing is essential to address the inherent mode-coupling. (*f*) FM-MCF but where each core supports a few modes offering the potential for ultrahigh levels of spatial channel count (highest so far 19 cores × 6 modes = 114 modes). Adapted from [5], fig. 2).

The range of potential technological SDM approaches is ultimately defined by fibre design and, as summarized in figure 5, several options are currently under investigation.

### (a) Fibre bundles

The first, and arguably most obvious approach, is to use an array of thin single-core fibres (figure 5*a*), possibly in some form of common polymer coating to improve rigidity and handling (see figure 5*b*-referred to as multi-element fibre (MEF) [18]). While a bundle approach offers significant merits in term of ease of practical system implementation, it offers only limited scope for increased spatial densities and associated device integration. The MEF approach does however offer some integration opportunities, particularly with respect to optical amplification [18]. Here, individual fibre elements, the majority containing a (single mode) erbium-doped core, can be embedded in a polymer coating material of lower refractive index than silica and the structure engineered to ensure that all neighbouring elements are in mutual optical contact. In this instance, one (or more if required) coreless elements can be used to deliver MM pump radiation from a low brightness but high-power MM pump diode into the bundle. The pump radiation rapidly spreads into the glass cladding of all other fibre elements where it is efficiently absorbed in those elements containing erbium-doped cores, leading to population inversion and hence gain for signals propagating within them. The multiple individual amplifiers share the pump radiation from a single common, relatively low-cost MM pump diode, moreover the pump combiner is effectively manufactured as part of the inherent fibre fabrication process, further reducing costs. Note that interfacing SSMFs to MEFs is also relatively straightforward: the common polymer jacket can simply be stripped away and splices made directly to the individual elements making for ready integration with existing SM components. Both transmission MEFs and MEF amplifiers have now been fabricated with as many as seven individual fibre elements and amplified transmission experiments have successfully been performed validating the basic principles of the approach [18]. The future challenges associated with the technique relate to reducing the propagation losses to levels comparable to SSMF (i.e. ensuring no additional loss due to the small element dimensions (typically approx. 80 μm), their close physical proximity and their local environment).

### (b) Multicore fibres

In multicore fibre (MCF), the distinguishable spatial pathways are defined by an array of physically distinct single-mode cores (figure 5*c*) integrated directly into the fibre cross-section. The revival in interest in using MCFs for data transmission took off following two separate papers on experiments on WDM data transmission in seven-core MCF in the Postdeadline Session of OFC 2011, with 56 Tbit s^−1^ capacity over 76.8 km [19] and 109 Tbit s^−1^ capacity over 16.8 km [20] reported by groups working in the USA and Japan, respectively. The cross-talk achieved in these fibres was sufficiently low (by virtue of the relatively large core spacing used) that the cores could be considered as effectively independent information channels over the associated propagation distances and for the particular modulation formats used. A later detailed study of the tolerance of various advanced modulation format signals to in-band accumulated cross-talk has shown that less than −25 dB cross-talk levels are typically required at the output of the transmission line to avoid significant penalties [21], providing insight into the levels of cross coupling per unit length required to transmit data over a given length scale.

To help reduce cross-talk still further, most subsequent MCFs have used more advanced core designs that incorporate trenches to better restrict the modal tails from extending too far into the cladding towards adjacent cores [22], and have used different geometric core arrangements to reduce the number of surrounding cores of similar design within the cross-section (e.g. a ring of cores of identical design such that each core has just two nearest neighbours rather than six for the central core in the hexagonal configuration used in these initial experiments [23]), or a hexagonal lattice of cores in which all nearest neighbours have a different design to restrict phase matching and hence to reduce cross-coupling (referred to as heterogeneous MCFs [24] as opposed to homogeneous designs where all cores are identical). Small variations in core properties along the fibre length can also help reduce distributed cross-coupling in both homogeneous and heterogeneous MCFs, as can the use of opposing signal propagation directions in adjacent cores (referred to as propagation direction interleaving [25]).

**Figure 6.**
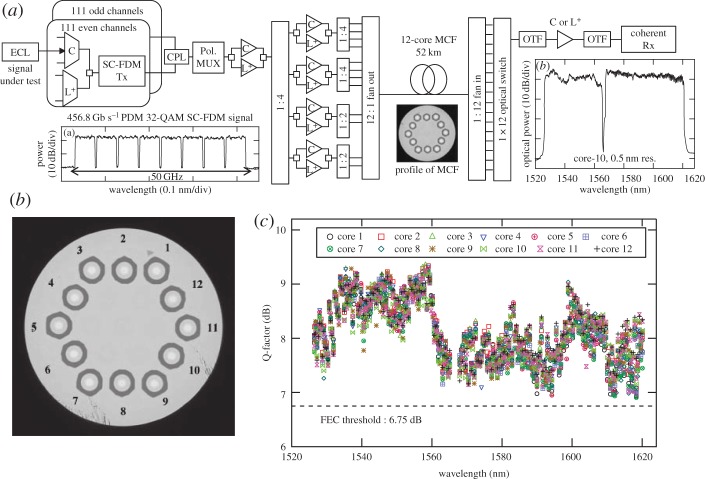
1.01 Pbit s^−1^ MCF WDM/SDM/PDM transmission experiment [23]. (*a*) Schematic diagram of the transmission system set-up, showing (from left to right) the modulation of the two sets of 111 channels, splitting and amplification for launch into the 12 : 1 fan-in device, transmission over the 52 km 12-core MCF, followed by the 1 : 12 fan-out device and a 1×12 optical switch to select one core for detection by the coherent receiver. ECL, external cavity laser; SC-FDM Tx, single-carrier frequency-division multiplexed transmitter; CPL, coupler; Pol Mux, polarization multiplexer; OTF, optical tuneable filter; Rx, receiver. (*b*) Microscope image of the cross-section of the one-ring, 12-core fibre. (*c*) Measured Q-factors of the 222 WDM channels in each of the 12 cores after 52-km transmission. Adapted from [5].

Homogeneous MCFs with a typical core spacing of approximately 30–40 μm (with a mode area well matched to that of SSMF) have been demonstrated, offering impressively low cross-talk levels (e.g. less than −90 dB km^−1^ for certain designs), thereby enabling independent parallel transmission over multi-1000 km length scales. Indeed, the highest capacities and longest transmission distances demonstrated in SDM system experiments to date have all used such independent core homogeneous MCFs. In particular, the first SDM experiments at the petabit/s capacity level (figure 6*a*) were achieved over a 52 km length of 12-core MCF [25], with the 12 cores arranged in a single ring (as shown in figure 6*b*). Two hundred and twenty-two WDM channels with 32-QAM modulation format signals at 400 Gb s^−1^ per channel were used to obtain an aggregate spectral efficiency of 91.4 bit^−1^ s^−1^ Hz^−1^ and both the C- and extended L-band (approx. 1530–1620 nm) were used in order to achieve a total capacity of 1.01 Petabit^−1^ s^−1^ (figure 6*c*). The first long-haul experiments at the Exabit.km s^−1^ level (over 7326 km) used a 7-core MCF [26]. A range of MCF EDFAs have also been developed (with core counts of up to 19) to support such long-haul transmission experiments. Both core and cladding-pumped embodiments, which are preferable from a cost perspective, have now been demonstrated with encouraging results achieved.

Fibre reliability issues, in particular susceptibility to fracture, mean that MCF diameters beyond approximately 250 μm are not considered practical, placing a relatively firm bound on the number of cores that can be incorporated in MCFs for long-haul transmission. The progress to date seems to indicate that the maximum number of independent cores that one can practically envisage using for long-haul transmission lies somewhere in the range 12–19, although fibres with as many as 30 single-mode cores have just recently been demonstrated exploiting heterogeneous core arrangements and impressively low levels of cross-talk of less than −40 dB over 100 km have been achieved [27]. Note that for many shorter distance applications, for example for use within data centres, higher core densities and core counts will certainly be viable.

### (c) Few-mode fibres

The situation with respect to channel definition is very different for mode division multiplexing (MDM) in few-mode fibre (FMF). A FMF is a MMF that has been designed to support a limited and controlled number of modes. The earliest work concentrated on the simplest FMF, which supports three distinct spatial modes (referred to as 3MF). The true modes of the fibre are the HE_11_, TM_01_, TE_01_ and HE_21_ vector modes; however, in practice the LP pseudo-mode basis (i.e. the LP_01_ and degenerate LP_11_ modes constructed from linear combinations of the true vector modes) is used since these modes are more readily excited and detected, providing a total of six polarization and spatial modes. In MDM, the distinguishable spatial data pathways are defined either by the particular modes supported by the fibre, or alternatively orthogonal combinations of these modes. Since the modes all have significant spatial overlap, the data signals are prone to couple randomly between spatial channels during propagation. It is generally necessary to correct for the impact of this coupling at the receiver end. In MDM, the individual modes accumulate differential mode group delays (DMGDs) during propagation and likely also differential modal loss or gain due to mode-dependent performance of the various in-line components (i.e. fibres, amplifiers, MUX/DEMUX, etc.). The energy of a given data symbol launched into a particular spatial channel consequently spreads out into adjacent symbol time slots as a result of mode coupling and modal dispersion, rapidly compromising successful reception of the information that it carries. To mitigate these linear impairments, DSP-based adaptive equalization using multiple-input multiple-output (MIMO) processing is required at the receiver. MIMO signal processing is in fact already widely used in current commercial coherent optical transmission systems to enable PDM. Correcting for polarization mode-coupling as needed for PDM requires a 2×2 matrix MIMO realization with four finite impulse response (FIR) filters. For an MDM system with M modes, the respective algorithms need to be scaled to 2M × 2M MIMO, requiring 4M^2^ adaptive FIR filters.

Interest in MDM in FMFs really took off at OFC 2011, as was the case for MCFs. Three papers were presented on data transmission in 3MF supporting the LP_01_ and degenerate LP_11_ modes. Per-channel rates of 100 Gb s^−1^ over the two spatial mode groups was achieved over 4.5 km [28] and 40 km [29], while 56 Gb s^−1^ signals in three modes were transmitted over 10 km [30] (the quoted bit-rates include polarization multiplexing). The three-mode experiment [30] was the first to demonstrate full use of all degrees of freedom afforded by the FMF (six spatial and polarization modes), with signal recovery via full coherent 6×6 MIMO, thus proving the viability of the approach.

**Figure 7.**
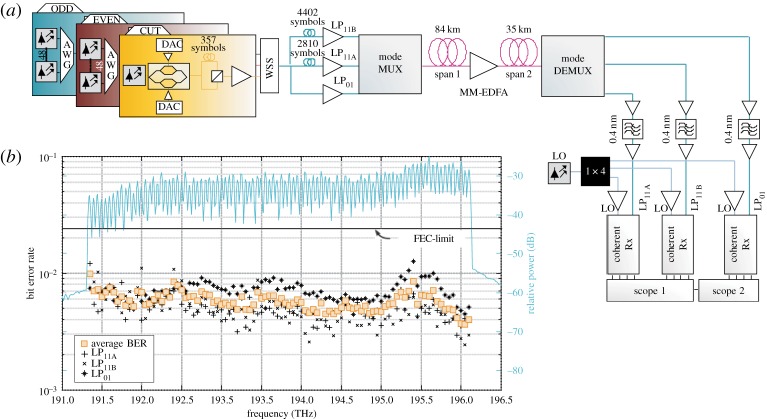
57.7 Tbit s^−1^ amplified WDM/MDM/PDM transmission experiment over a few-mode fibre [34]. (*a*) Schematic of the experimental set-up, showing (from left to right) the three sets of transmitters for odd and even channels and the channel-under-test, splitting and amplification for launch into the mode multiplexer, transmission over the two few-mode-fibre spans with in-line MM-EDFA, mode demultiplexer and simultaneous reception of the channels transmitted in the threemodes for MIMO processing. AWG, arrayed waveguide grating multiplexer; DAC, digital-to-analogue converter; WSS, wavelength-selective switch; LO, local oscillator. (*b*) Measured bit-error-rates (markers) of all 96 channels in each of the three modes and optical spectrum (blue curve) of all 96 channels after transmission over the 119 km of few-mode-fibre with a mid-span amplifier. Adapted from [5].

To fully compensate for the effects of DMGD and mode coupling using MIMO, the equalization filter length needs to be longer than the impulse response spread of an individual symbol. The DMGD of the LP modes in step-index core designs (as used in the first demonstrations of MDM in 3MF) is a few ns/km, meaning that the number of taps required for MIMO processing becomes increasingly challenging for transmission distances much above 10 km. Controlling the overall level of DMGD in MDM transmission fibres is therefore a primary design consideration and a large amount of work has been devoted to develop FMF core designs providing substantially reduced values (as well as on the DSP algorithms themselves). Using graded-index core designs featuring an accurately parabolic refractive index profile with an outer trench DMGD values of order 50 ps km^−1^ have been achieved for 3MF [31,32]. Moreover, such fibre designs also provide for low loss for each guided mode and low intrinsic mode-coupling (due to the relatively wide effective index spacing between all mode groups). Significantly, it has also now been demonstrated that DMGD compensation is possible by constructing transmission lines comprising lengths of FMFs with opposing signs of DMGD [31,33,34]. Link averaged net values of DMGD as low as approximately 5 ps km^−1^ have been achieved in this way. This opens up the possibility of transmission over more than 1000 km length scales when combined with the use of a suitable FM-EDFA [35]. To date, the highest net capacity transmitted via MDM over an FMF is approximately 57 Tb s^−1^ (after subtracting the overhead for FEC) [34]. Here, 96 WDM channels, each running at 200 Gb s^−1^, were transmitted over a 119 km MDM transmission line supporting three spatial modes, and using an inline 3M-EDFA (figure 7*a*,*b*). Scaling this DMGD compensation approach to a greater number of modes will be increasingly challenging and will require a greater number of different fibres within the link. Nevertheless, extension to 6MF has been shown to be viable, with transmission distances of several 100 km already demonstrated in recirculating loop experiments using a fully fiberized 6M-EDFA [36].

While low cross-talk and zero net DMGD are aspirational targets in terms of reducing the computational cost of MIMO processing, the optimum fibre parameters are likely to be defined in practice by other considerations. Indeed, many contend that since MIMO processing is almost unavoidable, the optics should be designed to take most advantage of this fact both in terms of offering new opportunities for low-loss spatial channel multiplexing, for example, allowing the use of photonic lanterns which naturally excite orthogonal groups of modes rather than distinct modes for use as spatial channels [37], and to reduce the effects of intermodal dispersion/gain/loss to maximum benefit. For example, for a system exhibiting weak mode-coupling then a given data symbol carried by multiple modes with different group indices will spread in time linearly with fibre length. However, if we use a fibre exhibiting strong mode-coupling, the temporal spread follows a random-walk process and this will scale with the square-root of fibre length [38]. Counterintuitively then perhaps, strong mode-coupling can potentially reduce the number of taps required for MIMO processing and consequently the DSP complexity. Indeed, this is analogous to the use of spinning to reduce PMD in current SMFs. Similarly, the impact of differential modal gain and loss can in principle be largely mitigated by strong mode-coupling over a suitable length scale relative to the amplifier spacing [39]. Similarly, instead of reducing DMGD to minimize a linear impairment (which can be compensated), it has been shown that a larger DMGD can reduce the more problematic intermodal nonlinear impairments [40]. The issue of nonlinear effects in FMFs is a complex problem and while considerable progress has been made much future effort will be needed to understand intermodal nonlinear penalties, particularly in the limit of many densely packed spatial channels.

The current challenge in MDM transmission is to scale the basic approach to a greater number of modes. Just recently results on nine LP-mode group fibres have been reported supporting a total of 15 distinct spatial modes, with transmission over a distance of 23.8 km obtained [41]. Work has even begun on using MDM for high-capacity transmission in conventional MMFs with core/cladding diameters of 50/125 μm and 62.5/125 μm. Such fibres support more than 100 modes; however, through careful mode excitation and filtering of the majority of HOMs it has proved possible to send data over a fixed number of the lowest order modes. To date, up to six spatial modes have been used, with more than 20 Tbit s^−1^ transmission demonstrated over 17 km of fibre [42]. How far it will be possible to go in terms of number of modes and associated transmission distance is yet to be determined and will certainly be constrained by the rapid rise in MIMO complexity as these parameters are increased.

### (d) Combining multicore and few-mode fibre approaches

In the above discussions, we have described the basic approaches to SDM as distinct; however, the current frontier in SDM research is to combine multiple SDM approaches to achieve much higher levels of spatial channel count (referred to as channel multiplicity). For example, by bringing the cores of a MCF closer together to ensure strong linear mode-coupling, it is possible to establish supermodes defined by the array of cores. These supermodes can then be used to provide spatial information channels for MDM to which MIMO can be applied [43]. Such fibres are referred to as coupled-core fibres (figure 5*e*) and they enable significantly higher spatial channel densities than can be obtained using isolated core MCF designs. In this limit, one may perhaps argue that the MCF is effectively a form of FMF, albeit one in which the modal properties can be engineered through the geometric arrangement of coupled cores.

**Figure 8.**
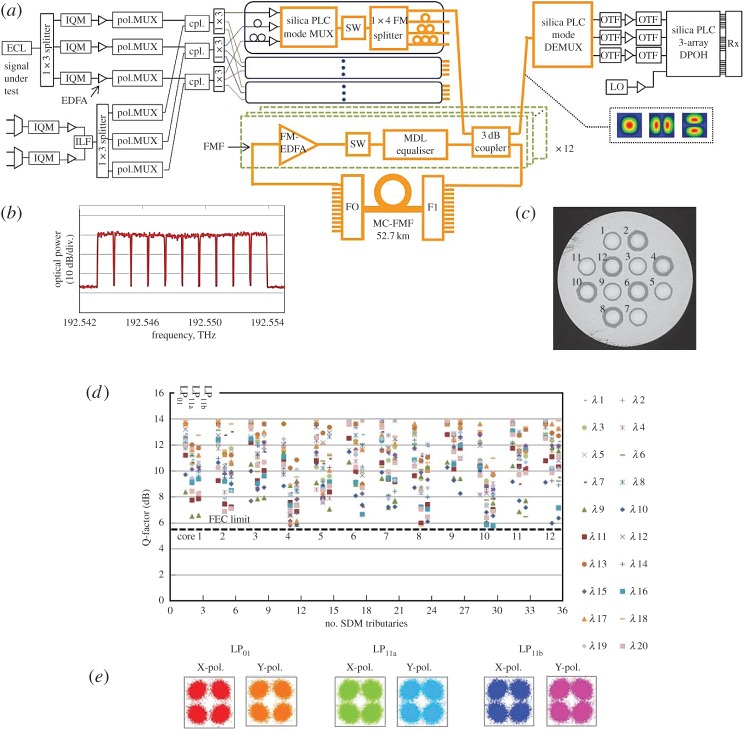
Thirty-six channel FM-MCF transmission over 527 km. (*a*) Experimental set-up, (*b*) low-baud rate multi-carrier signal, (*c*) cross-section of low-DMD heterogeneous 12 core × 3 mode MC-FMF, (*d*) Q-factor measurement results after 527-km transmission, (*e*) constellations of core no.11, wavelength no.10, subcarrier no.4. (Figure courtesy of T. Mizuno of NTT Laboratories, Japan).

More recently however the possibility of using the MCF approach with *N* independent cores but where each core supports *M*-modes rather than just 1 has received much attention. Such fibres are referred to as few-mode multicore fibres (FM-MCFs; figure 5*f*) and they support a total of *M*×*N* spatial channels, with MIMO required on a per-core basis. Impressive progress has been made and just recently data transmission at a record spectral efficiency of 345 bit s^−1^ Hz^−1^ was reported through a 9.8 km FM-MCF containing 19 cores with each core supporting six spatial modes, providing a total of 114 distinguishable spatial channels [44]. Longer distance data transmission in FM-MCFs has also been reported—the best result to date being 20 WDM channels, each supporting 40 Gbit s^−1^ PDM-QPSK transmission over 527 km of a FM-MCF containing 12 cores, each core guiding three spatial modes (i.e. 36 SDM channels in total) [45] (see figure 8 for a detailed schematic of the experimental test bed, cross-sectional image of the fibre and associated transmission results).

## 4. Space division multiplexing technology and integration

While the development of innovative fibres for SDM goes on, much work has focused on the component and connectivity challenges that are essential to building cost-effective systems around SDM fibres [46]. The development of integrated transmitter and receiver arrays along with compatible fibres and components is essential, as was historically the case in datacomms [47]. The availability of compact, low-loss spatial channel Mux and Demux devices in particular is critically important for any SDM approach. Excellent progress has been made in recent years for both MCF and FMF implementations in the context of both fibre-based and integrated solutions (e.g. three-dimensional direct write waveguides and photonic lanterns [48], mode selective couplers [49] and integrated silicon devices [50]). The development of SDM amplifiers is key for long-haul SDM applications and has received significant attention. The potential cost and power savings associated with cladding-pumped SDM amplifiers of different forms are particularly appealing [51]; however, ensuring low channel-dependent gain and high efficiency requires complex fibre designs (particularly in the case of FMFs where all the modes propagate through the same inverted ion population), moreover the noise figure/spectral operating ranges are likely to be compromised by the relatively low brightness of the multimode semiconductor pump lasers used. Cladding pumping also inevitably provides far less options for independent channel gain control and provides further mechanisms (e.g. cross-spatial channel gain saturation) for inter-spatial channel effects. Much further work is thus required to establish the capability and practicality of the various forms of SDM amplifier. Without such amplifiers, the attraction of SDM certainly diminishes. Likewise, SDM ROADMs are considered to be an essential component with potential for cost saving benefits. However, the design of these can become quite complicated depending on the choices made regarding the type of superchannels to be used (e.g. frequency or spatial [52]) and the strategy for switching, e.g. wavelength by wavelength, all modes together, or mode-by-mode, with mode interchange.

**Figure 9.**
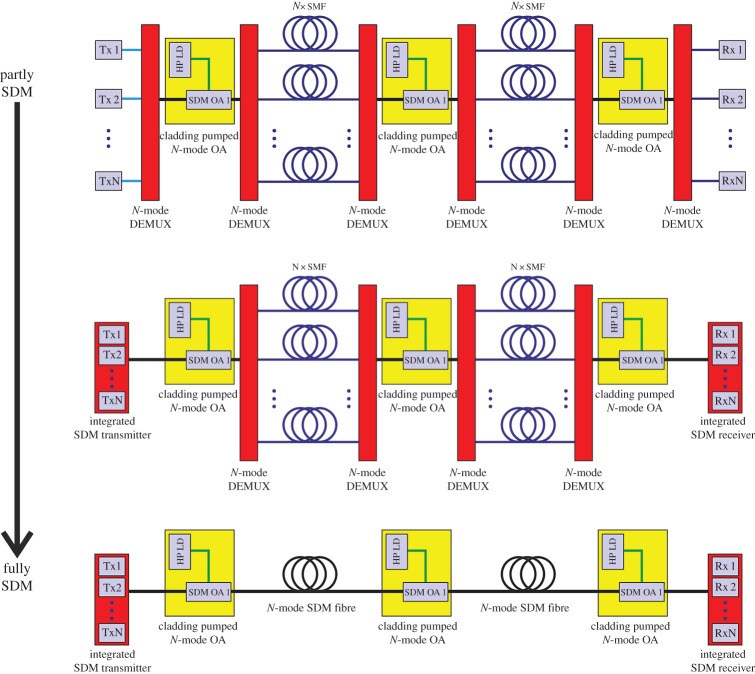
One possible upgrade scenario from partly SDM systems to fully SDM long-haul systems. In the first instance, SDM compatible components, such as amplifiers, may be incorporated into otherwise conventional SSMF systems to provide cost saving benefits. As additional components become available, e.g. SDM transceiver arrays, these also may start to be integrated to drive down system costs and to improve performance. Only once these subsystems have been proved and the installation of new fibres becomes unavoidable will adoptionof integrated SDM transmission systems be seriously considered.

Even if it proves possible to demonstrate cost reduction benefits through SDM, it is unrealistic to imagine that fully SDM systems will be installed immediately. Figure 9 illustrates one possible adoption path where it is anticipated that SDM components (e.g. amplifiers) will first be installed as upgrades to the existing single-mode infrastructure to realize short-term cost-savings, and to prove the technology. As further integrated components are developed (e.g. transmitters), these will also likely be incorporated to further reduce costs. Only once confidence has been achieved in all aspects of the technology is it conceivable that fully SDM systems incorporating SDM transmission fibres might be deployed.

## 5. Conclusion

Over the past 5 years, major progress has been made in developing new fibre technologies for high-capacity communications. Gradual improvements are being made in SSMFs in terms of effective area and loss reduction, new broadband amplifier options are emerging and steady progress is being made in the area of hollow core fibres. The primary advances though have been in the area of SDM, especially in the development of high-performance MCF, FMF and MC-FMFs, as well as the associated components required to launch and amplify the individual spatial channels. As a result, various experiments have reported record per-fibre capacities and capacity length products. Initial demonstrations of switching/routing and associated networking have also now been performed. However, the research is still very much in the exploratory phase with much further work needed to show whether significant cost-per-bit reductions can be achieved at levels of per-channel reliability and performance that are competitive with existing single-mode fibre technology. Photonic integration will be absolutely essential in realizing cost reduction through SDM and this work is really still at a very early stage. Nevertheless, the results to date indicate that there is the potential to achieve a 10–100-fold improvement in per-fibre capacity, at least in the laboratory. Whether this will ever translate into commercial long-haul systems is yet to be seen, though there is already evidence that some of the technology may find use in the shorter term in short reach applications, for example in Data Centres, where achieving high spatial path densities is critical and the barrier to entry for new technologies is very much lower than for long-haul communication networks.

## Data accessibility

Data contained in this paper is openly available from the University of Southampton repository at http://dx.doi.org/10.5258/SOTON/383293.
